# Heterogeneous effects of spatially proximate firearm homicide exposure on anxiety and depression symptoms among U.S. youth

**DOI:** 10.1016/j.ypmed.2022.107224

**Published:** 2022-08-25

**Authors:** Shani A.L. Buggs, Xiaoya Zhang, Amanda Aubel, Angela Bruns, Nicole Kravitz-Wirtz

**Affiliations:** a Violence Prevention Research Program, Department of Emergency Medicine, University of California, Davis School of Medicine, Sacramento, CA, USA; b Department of Human Ecology, University of California, Davis, Davis, CA, USA; c Department of Sociology & Criminology, Gonzaga University, Spokane, WA, USA

**Keywords:** Firearm violence, Community violence, Race-ethnicity, Youth, Mental health, Violence exposure, Homicide

## Abstract

The burden of firearm homicide in the United States is not evenly distributed across the population; rather, it disproportionately affects youth in disadvantaged and marginalized communities. Research is limited relevant to the impacts of exposure to firearm violence that occurs near where youth live or attend school – spatially proximate firearm violence – on youths' mental health and whether those impacts vary by characteristics that shape youths' risk for experiencing that exposure in the first place. Using a dataset linking the Fragile Families and Child Wellbeing Study with the Gun Violence Archive (N = 3086), we employed propensity score matching and multilevel stratification to examine average and heterogeneous associations between spatially proximate firearm homicide exposure and anxiety and depression among all youth and then separately for boys and girls. We found a statistically significant average association between firearm homicide exposure and symptoms of depression among youth. Furthermore, heterogeneous effects analyses yielded evidence that the average association is driven by youth, and particularly boys, who are the most disadvantaged and have the highest risk of firearm homicide exposure. The results of this study suggest that the accumulation of stressors associated with structural disadvantage and neighborhood disorder, coupled with exposure to spatially proximate and deadly firearm violence, may make boys and young men, particularly Black boys and young men, uniquely vulnerable to the mental health impacts of such exposure. Ancillary analyses of potential effect moderators suggest possible future areas of investigation.

## 1. Introduction

Firearm homicide is one of the most devastating events to impact not only individuals but entire communities. In the United States (U.S.), between 2010 and 2020, firearm homicide prematurely ended the lives of >150,000 people, including approximately 9400 young people under the age of 18 (Centers for Disease Control and Prevention, 2022). Like nearly all poor health outcomes in the U.S., the burden of firearm homicide is not evenly distributed; rather, it is endured disproportionately by minoritized and marginalized communities. Latinx children and teens experience firearm homicide at 3 times the rate of their white counterparts. It is the 2nd leading cause of death for Black girls and women ages 15–24 and has been the leading cause of death for Black boys and men ages 15–24 for over 30 years (Centers for Disease Control and Prevention, 2022). The inequitable distribution of community firearm violence and, by extension, its myriad social and health repercussions, is a catastrophic yet predictable outcome of decades of structural racism, state-sanctioned violence, and disinvestment in institutions in racially and economically segregated neighborhoods that are critical to health and safety.

Homicide is arguably the most disastrous outcome of firearm violence, but it is the tip of the iceberg. Nonfatal firearm injuries occur up to 4 times as frequently as homicide (Hipple et al., 2020; Kaufman et al., 2021), and studies find anywhere from 13% to 65% of young people report hearing gunshots or witnessing someone get shot or shot at in their lifetimes (Bancalari et al., 2022; Finkelhor et al., 2015; Fitzpatrick, 1997; Stein et al., 2003; Turner et al., 2019; Zimmerman and Posick, 2016). Community violence exposure is associated with a host of emotional and behavioral problems, including posttraumatic stress symptoms, anxiety, and depression (e.g., Ahern et al., 2018; Berman et al., 2021; Foell et al., 2021; Gaylord-Harden et al., 2011; Gorman-Smith and Tolan, 1998), particularly when firearms are involved (Aubel et al., 2021; Fowler et al., 2009; Kagawa et al., 2020; Opara et al., 2020; Ranney et al., 2019; Rowhani-Rahbar et al., 2019). Whereas the majority of these studies define “exposure” as being a victim, perpetrator, or witness to violence (Cimolai et al., 2021; Mitchell et al., 2021; Shulman et al., 2021; Spano and Bolland, 2013; Turner et al., 2019), the impacts of firearm violence can also affect others in the community, regardless of direct or witnessed experience.

Broader exposure to spatially proximate firearm violence – any incident that occurs near where someone lives or spends time – is both widespread and uneven, further reflecting structural inequities that disproportionately disadvantage and compromise the health and well-being of Black and Latinx youth. One study found 56% of Black youth and nearly half of Latinx youth in large U.S. cities live within 1300 m (approximately 0.8 miles) of a past-year firearm homicide occurrence, with 1 in 4 Black youth and 1 in 5 Latinx youth experiencing 3 or more incidents; comparable percentages for white youth were 17% for any incident and <1% for 3 or more (Kravitz-Wirtz et al., 2022). In a separate analysis using the same dataset, Black and Latinx youth in middle-to-high income households were nearly twice as likely as white youth in low-income households to live or attend school near a deadly firearm violence incident (James et al., 2021). These incidents of spatially proximate firearm violence can become salient in youths' daily lives via numerous sources, including learning about an incident from peers, family members, or media; hearing or seeing police, ambulances, or crime scenes; or passing memorials or vigils where violent deaths have occurred (Wintemute et al., 2022).

A small but growing number of studies have investigated associations between spatially proximate firearm violence exposure and youth mental health outcomes. One study found an increase in emergency department utilization for mental health-related symptoms among children in the days following a firearm violence incident occurring within a few blocks of their homes (Vasan et al., 2021). Another study found that every additional exposure to a deadly firearm violence incident near a child's home in the past year increased behavioral problems by nearly 8%, even after accounting for socioeconomic indicators and area-level crime (Gard et al., 2022). However, few published studies have specifically explored the effects of spatially proximate firearm violence exposure on anxiety and depression among young people, and even fewer have analyzed potential variation in those effects. One study of juvenile system-involved adolescent males found that recent exposure to firearm violence (experienced or witnessed a shooting) was associated with increased levels of aggression and anxiety, with less support for increased depression (Shulman et al., 2021). Another study found that the occurrence of a past-year firearm homicide within 1 mile of an adolescent's home or school was associated with significantly worse symptoms of anxiety and depression for girls but only marginally worse symptoms of anxiety for boys (Leibbrand et al., 2020).

When researchers have explored heterogeneity in the effects of spatially proximate firearm violence exposure on youths' mental health, they have typically focused on variations by gender and race/ethnicity, rather than also considering modifiable social determinants that could additionally affect the exposure-outcome relationship, such as neighborhood disadvantage or prevalence of community violence. As a result, much remains unknown about whether the impacts of spatially proximate firearm violence exposure on youths' mental health vary by the same community-level characteristics that shape youths' risks of experiencing that exposure in the first place, and what risk and protective factors may underlie such variation. It is possible that further stratification may reveal heterogeneous effects in this relationship, suggesting that more tailored interventions are necessary to most effectively address mental health-related harms related to firearm violence exposure.

This study expands existing literature on the association between spatially proximate firearm violence exposure and youths' mental health outcomes – specifically, anxiety and depression –by examining average and heterogeneous effects using quasi-experimental methods. We use a rich, longitudinal dataset that allows for the inclusion of a comprehensive array of individual, familial, and neighborhood-level characteristics that contribute to systematic differences in youths' probability of spatially proximate firearm violence exposure. In turn, we employ propensity score matching methods to approximate causal effects of such exposure on youths' anxiety and depression symptoms. Further stratifying by youths' propensity of exposure helps address theoretical debates about the role of firearm violence exposure as an acute stressor among youth with relatively low risks of exposure or one of many cascading chronic stressors among youth with relatively high risks of exposure. Consequently, we shed light on the ways youths' risks of exposure to spatially proximate firearm violence may also shape their varying responses to such exposure. This approach can also suggest malleable social-ecological factors that may increase youths' vulnerability or resilience to the effects of exposure, offering important insights for the development of focused strategies to disrupt cycles of violence and trauma.

## 2. Methods

### 2.1. Data and variables

#### 2.1.1. Sample

In this study, we used data from waves 5 and 6 of the Fragile Families and Child Wellbeing Study (FFCWS) merged with incident-level data on the precise location and date of deadly interpersonal firearm violence incidents (hereafter, firearm homicides^1^) from the Gun Violence Archive (GVA). FFCWS is a birth cohort study following a stratified, multistage, probability sample of children born between 1998 and 2000 in 20 large US cities, with an oversample of births to unmarried parents (Reichman et al., 2001). Data from the GVA, a national open-source database of firearm violence incidents for years 2014 onward, were linked to FFCWS based on the latitude and longitude coordinates of children's home and school addresses. Children (hereafter, youth) were approximately 15 years of age at the time of data linkage.

In addition to the wide array of individual, familial, and neighborhood-level health and social indicators from FFCWS, the merged dataset provides information on the total number of firearm homicides occurring within cross-classified categories of distance from youths' homes or schools (between 100 m and 1 mile) and time before their wave 6 data collection date (between 7 days and 1 year). The analytic sample for this study included 3086 of 3444 youth who participated in wave 6 data collection. We excluded 358 observations that were missing important covariates (e.g., internalizing or externalizing behavior) in wave 5. Missing data for other variables at waves 5 and 6 were imputed 20 times using multiple imputation by chained equations with the MICE package in R (van Buuren and Groothuis-Oudshoorn, 2011).

#### 2.1.2. Predictor

The main exposure of interest was spatially proximate, past-year firearm homicide occurrence. We created a dichotomous measure of exposure indicating whether at least 1 firearm homicide occurred within 1300 m (Kravitz-Wirtz et al., 2022) of youths' homes or schools during the 365 days before their wave 6 data collection date. We examined firearm homicides occurring proximate to youths' homes and/or schools because youths' homes and schools were often within our same exposure radius (<1207 m on average).

#### 2.1.3. Outcomes

We utilized 2 measures of youths' mental health. First, depression was measured using 5 items adapted from the Center for Epidemiologic Studies Depression Scale (CES—D), which describes youths' feelings in the past 4 weeks and uses a 4-point rating scale ranging from *1 = strongly agree* to *4 = strongly disagree* (i.e., “I feel I cannot shake off the blues, even with help from my family and friends;” “I feel sad;” “I feel happy;” “I feel life is not worth living;” and “I feel depressed”). We reverse-coded each item (except “I feel happy”) so that higher values represented greater symptoms of depression, summed the scores on each item, and then standardized the total score to have a mean of 0 and a standard deviation of 1.

Second, anxiety was measured using 6 items adapted from the Brief Symptom Inventory 18 (BSI 18) anxiety subscale, which similarly describes youths' feelings in the past 4 weeks and uses a 4-point rating scale ranging from *1 = strongly agree* to *4 = strongly disagree* (i.e., “I have spells of terror or panic;” “I feel tense or keyed up;” “I get suddenly scared for no reason;” “I feel nervous or shaky inside;” “I feel fearful;” and “I feel so restless I can't sit still”). We reverse-coded each item so that higher values represented greater symptoms of anxiety, summed the scores on each item, and then standardized the total score to have a mean of 0 and a standard deviation of 1.

#### 2.1.4. Covariates

We carefully selected multiple individual, familial, and contextual factors as covariates in our analyses given their association with the exposure and/or outcomes. These variables included individual demographic (age, gender, race/ethnicity), family socioeconomic (mother's educational attainment at youth's birth), and neighborhood (disadvantage, collective efficacy) characteristics, other exposures to violence (peer bullying, secondary exposure to intimate partner violence or community violence via the primary caregiver), as well as behavioral characteristics of youth (impulsivity, internalizing behavior, externalizing behavior) and of mothers or primary caregivers (depression, substance use). All covariates were measured at baseline (birth) or wave 5 (except neighborhood characteristics, which were measured at wave 6), prior to the exposure and outcomes.

### 2.2. Statistical analysis

Our analytic strategy is detailed in the Appendix (Supplementary Analysis Details) and summarized here. To estimate the effects of firearm homicide exposure, we first used covariate-adjusted ordinary least squares (OLS) regression, averaged across the imputed datasets, and then employed propensity score matching methods to approximate causal effects. We calculated the predicted probability (propensity or risk) of exposure for each observation and estimated the average effects of exposure on anxiety and depression for matched treated (exposed) youth. Next, we examined the heterogeneous relationships between spatially proximate firearm homicide exposure and youths' mental health using the stratification-multilevel method (Xie et al., 2012), which groups youth into propensity score strata representing different levels of risk of experiencing at least 1 firearm homicide (ranging from low to high). This approach allows for the estimation of stratum-specific effects of firearm homicide exposure on youths' mental health.

Finally, to examine possible sources of heterogeneous exposure effects, we conducted ancillary analyses comparing the mean values of additional variables across treated and control youth within each propensity score stratum. These potential moderating variables included measures of youths' contact with and treatment by police, legal system involvement from arrest to detention, connectedness at home and school, and extracurricular and community involvement. Given data limitations that prevent precise determination of the temporal ordering of events, we were unable to perform formal tests of moderation. Rather, these ancillary analyses were intended as exploratory and hypothesis-generating assessments of factors that may exacerbate risk or confer resilience among youth who may be differentially vulnerable to the mental health impacts of firearm homicide exposure.

Statistical analyses were conducted for the full sample and separately for boys and girls using Stata, version 16 (StataCorp, 2017). The Institutional Review Boards at the University of California, Davis and Gonzaga University each determined that this analysis of secondary, deidentified data was exempt from human subjects review.

## 3. Results

### 3.1. Average associations between firearm homicide exposure and youths' depression and anxiety symptoms

Table 1 presents estimates of the average effects of spatially proximate, past-year firearm homicide exposure on youths' depression and anxiety symptoms using multivariate OLS regression (“unmatched”) and propensity score matching for all youth and then separately for boys and girls. In the unmatched analyses, the effect of firearm homicide exposure on depression symptoms was statistically significant for the full sample and marginally significant for girls. When matching was applied, the effect on depression remained significant only for the full sample. No statistically significant effects were found for anxiety using either analytic approach.

### 3.2. Heterogeneous associations between firearm homicide exposure and youths' depression and anxiety symptoms

To explore variations in anxiety and depression symptoms across youths' risk of spatially proximate firearm homicide exposure, youth were stratified by propensity scores according to their likelihood of exposure, with Stratum 1 representing the lowest risk of exposure and Stratum 3 representing the highest exposure risk. Tables 2a, 2b, and 2c, respectively, describe the characteristics of a typical youth in each stratum in the full sample, boys-only sample, and girls-only sample. The most striking difference across strata was related to racial/ethnic categorization. In the full-sample (Table 2a) and girls-only (Table 2c) analyses, a single-digit percentage of youth in Stratum 1 identified as Black, compared with 50% in Stratum 2 and approximately 75% in Stratum 3; approximately 60% of youth in Stratum 1 and 1% in Stratum 3 identified as White, while about 20% of youth in Strata 1 and 3 identified as Latinx. On the other hand, in the boys-only (Table 2b) analysis, 0% of the populations in Strata 1 or 2 identified as Black, while 91% of the Stratum 3 population identified as Black; in Stratum 3, <1% identified as White and 8% identified as Latinx.

Overall and by gender, youth in Stratum 3 were more likely than those in Stratum 1 to have both higher levels of factors associated with spatially proximate firearm homicide exposure risk and lower levels of factors associated with protection from that risk. For example, compared to those in Stratum 1, higher frequencies of Stratum 3 youth in the full-sample analysis had secondary exposures of community violence via caregivers who witnessed someone attached with a weapon or shot in the past year, agreed that gangs are a problem in their neighborhood, experienced neighborhood disadvantage, and reported lower levels of neighborhood collective efficacy (Table 2a). They also had higher measures of individual-level characteristics that are associated with anxiety and depression, including higher measures of impulsivity, internalizing and externalizing behaviors, and peer bullying.

Tables 3a, 3b, and 3c, respectively, show the heterogeneous effects of spatially proximate firearm homicide exposure on anxiety and depression symptoms for the full sample and for the boys-only and girls-only samples. Across all samples and propensity score strata, no statistically significant associations were found between exposure and anxiety. However, for the full sample, exposure was significantly associated with depression for youth in Stratum 3 (b = 0.132, se = 0.066) (Table 3a) – youth with the highest risk of firearm homicide exposure. Exposure was also significantly associated with depression among boys in Stratum 3, implying that the associations observed for the full-sample are driven by boys in the study (Table 3b). Boys in Stratum 3 with firearm homicide exposure had depression symptoms >20% of a standard deviation higher than their counterparts who were not exposed (b = 0.218, se = 0.103). The girls-only analyses of heterogeneity yielded no statistically significant associations between firearm homicide exposure and either depression or anxiety for any of the strata (Table 3c). No significant linear trends in the association across strata were found in any of the analyses, implying no detectable linear dose-response relationship from strata to strata.

### 3.3. Potential moderating factors for heterogeneous associations between firearm homicide exposure and youths' anxiety and depression symptoms

Ancillary analyses examined several potential moderating factors that may help account for stratum-specific effect heterogeneity, by comparing levels of each potential moderator among treated and control youth within and across strata. Significant associations suggest risk or buffering factors for further consideration.

Results from the boys-only analyses are presented in Table 4; tables from the full-sample and girls-only analyses are included in the Appendix (Supplementary Tables 1a and Table 1b). Boys in Stratum 3 who were exposed to firearm violence had significantly higher prevalence of witnessing police stops in the neighborhood than their counterparts who were not exposed (Table 4). They more frequently witnessed, knew someone who experienced, or themselves experienced unjust treatment by police. Being involved in extracurricular and community activities was marginally more prevalent among boys with (versus without) firearm homicide exposure in Stratum 3.

The mean values of the potential effect moderators for each stratum, as well as the percent difference between each stratum's means, are listed for all 3 samples in the Appendix (Supplementary Tables 2a, 2b, and 2c). Patterns across the strata were most similar in the full-sample and boys-only analyses; as risk of firearm violence exposure increased from Stratum 1 to Stratum 3, the mean values of potential moderators tended to reflect worse outcomes, and the percent differences between the lowest (Stratum 1) compared to the highest (Stratum 3) risk of exposure were often statistically significant. For example, between Stratum 1 and Stratum 3 in the full-sample analysis (Supplementary Table 2a), closeness of mother/caregiver-child relationships, connectedness at school, and extracurricular and community involvement significantly decreased, while the frequencies of youth encountering police, witnessing or experiencing unjust treatment by police, or having legal system involvement significantly increased.

## 4. Discussion

This study investigated the average and heterogeneous associations of spatially proximate, past-year firearm homicide exposure with anxiety and depression symptoms among relatively socioeconomically disadvantaged youth in large U.S. cities (Reichman et al., 2001). Given that structural and economic inequities correlate with elevated levels of firearm violence, these youth are also at a higher risk of exposure. Yet we found that even among this population, the effects of firearm homicide exposure were not evenly distributed. Using propensity score matching, we found a statistically significant average association between spatially proximate firearm homicide exposure and symptoms of depression among youth. However, heterogeneous effects analyses yielded evidence that this average association was driven by youth, and particularly boys, who were the most disadvantaged and had the highest risk of firearm homicide exposure; no statistically significant effects were observed for youth at relatively lower risks of exposure. This finding suggests that the accumulation of stressors associated with structural disadvantage, coupled with exposure to firearm violence, may make boys and young men uniquely vulnerable to the mental health impacts of such exposure. When considered alongside research showing that youth experiencing more complex adversity histories may be more susceptible to indirect firearm violence exposure, and that youth with a higher sense of safety are less likely to experience extreme sadness following exposure (Mitchell et al., 2021), our findings underscore the urgent need to implement and adequately fund community-based violence interventions, including but not limited to Advance Peace (Corburn et al., 2021; Corburn et al., 2022), Becoming a Man (Prochaska, 2013), and hospital-based violence interventions (Wical et al., 2020) that simultaneously offer young people access to mental health and coping support while also notably reducing all forms of community firearm violence. Our findings also point to the need for tailored interventions aimed at identifying and engaging youth at greatest risk of firearm violence exposure in affordable, accessible, and culturally-responsive mental health support services in their schools and communities. Furthermore, they highlight the harm to these vulnerable populations exacerbated by our societal failure to redress persistent structural inequities, including concentrated disadvantage, structural racism, and concentrated risk of firearm violence exposure, that can have devastating effects on physical and mental health and well-being.

Our findings specific to Black boys are notable and deserve further comment. In the boys-only analyses, those with the highest risk of exposure were overwhelmingly Black; in fact, there were no Black boys in either of the two lower risk strata. The finding that spatially proximate firearm homicide exposure was significantly associated with depression only among boys in the stratum with the highest exposure risk underscores both the disturbingly high risk of community firearm violence exposure among Black boys in the U.S. and the devastating toll that exposure can have on their mental health. While a number of researchers have examined the relationship between community violence exposure and adverse mental health consequences among Black boys (e. g., Gaylord-Harden et al., 2016; Gorman-Smith and Tolan, 1998; Lambert et al., 2021), findings have been inconsistent, with some detecting an association and others not. However, few have isolated the impact of deadly firearm violence exposure, which may have more harmful consequences on the mental health of youth and on socioeconomically disadvantaged Black boys in particular. This is a critical area of future study. Black boys in the U.S. currently lack easily accessible interventions for addressing poor mental health outcomes such as depression, anxiety, substance use disorders, and posttraumatic stress disorder symptoms like hypervigilance and emotional detachment, yet they endure distinct stressors related to racial discrimination, increased risk of state surveillance, contact and violence, and increased exposure to community firearm violence (Burrell et al., 2021). They also face particular barriers associated with receiving appropriate care even when they have access to mental health professionals; numerous researchers have noted concerns regarding overdiagnosis, underdiagnosis, and decreased likelihood of psychiatric treatment for African-American youth, as well as the likely role this misaligned care plays in fueling disparities in Black youth rates of school suspensions, expulsions, and contact with the juvenile and criminal legal systems (Baglivio et al., 2017; Holden et al., 2012; Liang et al., 2016; Marrast et al., 2016).

Furthermore, while outside the scope of the current study, we cannot ignore the necessity for more research examining any relationship between community firearm violence exposure and the alarming and unprecedented rise in firearm suicide among Black youth over the past 20 years (Sheftall et al., 2022). Scholars have noted that among Black youth, knowledge of family and peers' community violence exposure is associated with subsequent increases in suicide ideation (Lambert et al., 2021), further accentuating the need for more research to better understand these associations, especially those related to firearm-specific violence exposure.

As supported by our ancillary findings, protective factors and interventions for Black youth exposed to community violence include strengthening social ties and cohesiveness between youth and their formal or surrogate caregivers (Hammack et al., 2004), increasing social support, lowering social constraints for discussing violence, and increasing connectedness with teachers in school (Ozer and Weinstein, 2004). Future research and practice should consider whether interventions prioritizing these domains may help mitigate the negative mental health impacts of firearm-specific violence exposure. However, we must not further delay implementing culturally-affirming and healing-centered responses to community firearm violence and its mental health sequalae for Black youth (particularly boys) with chronic exposure. Trusted and credible community leaders and peers could be trained on violence intervention and therapeutic modalities suitable and acceptable to Black youth and their families, and parents and teachers could be educated on strategies that strengthen their connections and cohesiveness with Black youth in their lives. Furthermore, data on community firearm violence could be leveraged to identify and deliver therapeutic services in near-real time to the youth who may need it most.

Neither the average nor heterogeneous effects analyses yielded statistically significant relationships between firearm homicide exposure and anxiety, although the direction and magnitude of the estimates in the full-sample and girls-only analyses suggest a possible association may be observed with a larger pool of youth. Using the same dataset as in this study, but without propensity score matching, Leibbrand et al. (2020) found evidence that spatially proximate firearm homicide exposure is significantly associated with anxiety and depression among girls but not boys. When the researchers applied survey weights in supplementary analyses, anxiety and depression were no longer significantly associated with the exposure among girls, yet depression became significant among boys. While our propensity score matching approach helps address sampling design limitations and the differential likelihood of community violence exposure, the stratification method constrained the within-stratum sample size and power available for analyses. Thus, though we did not observe a significant association between spatially proximate deadly firearm violence exposure and anxiety, we have reason to believe that a true association may still exist. Future examinations of this relationship utilizing larger datasets should also incorporate exposure to spatially proximate nonfatal firearm violence, given the higher frequency of nonfatal (versus fatal) firearm injuries in the U. S.

While the results from our ancillary analyses of potential moderators were hypothesis-generating, rather than hypothesis-testing, they offer additional insights into interesting areas for future exploration. In the full-sample and boys-only analyses, youth at high risk of exposure to spatially proximate firearm homicide and who were indeed exposed had higher prevalence of experiences with police stops and unjust treatment by police than their counterparts with similar risk of firearm homicide exposure but who were not exposed. The fact that these young people were more likely to have witnessed police stops is not surprising on its own, given that they also lived or attended school near a past-year deadly firearm homicide incident. However, the increased risk for experiencing depression following the exposure, coupled with the increased probability of police contact, may warrant additional analysis. Studies have shown that youth of color are more likely than white youth to experience emotional distress during witnessed police stops (Jackson et al., 2021), and that persistent police contact has adverse mental health outcomes for Black youth (Jindal et al., 2022). Research has also found that youth reporting personal or vicarious police stops have worse self-reported health than those with no police contact, and that perceived procedural injustice by the police exacerbates the association between police contact and self-reported health (McFarland et al., 2019). Taking our findings together with existing research, it is recommended that interventions aiming to reduce community firearm violence exposure while simultaneously reducing youth contact with police, especially in areas of high disadvantage and high risk of exposure, receive greater attention and consideration.

We also observed a potential moderating effect of extracurricular and community involvement on the association between firearm homicide exposure and depression for boys with the highest risk of exposure, with those exposed to firearm violence having marginally higher extracurricular and community involvement than those who were unexposed. It is possible that boys with greater extracurricular and community involvement are most affected when firearm homicide occurs within their communities, as they may be more likely to have social ties to the victim or connection to community. Alternatively, it is possible that despite their involvement in extracurricular and community activities, boys living in these highly vulnerable environments are at remarkably higher risk of depression when firearm violence occurs. Again, though, as this was not a formal test for moderation, these results are merely suggestive of possible future areas of investigation.

### 4.1. Strengths and limitations

This study relies on a unique combination of population-representative, individual-level survey data on youth that has been geospatially linked with fine-grained information on incidents of deadly firearm violence. By leveraging the breadth of data available in the FFCWS and utilizing propensity score matching and stratification methods, we were able to not only analyze the impacts of spatially proximate firearm homicide exposure on youths' mental health, but to also conduct unprecedented examination of exposure effect heterogeneity, upon which firearm violence intervention and prevention strategies can build.

However, this research is not without limitations. First, observed associations may result from unmeasured factors that could render the heterogeneous associations nonsignificant. We attempted to minimize potential bias through our matching and analytic strategies, but unobserved confounders may nonetheless still exist. Also, findings must be considered within the context of the FFCWS, which oversampled unmarried parents and includes, on average, more socioeconomically disadvantaged families than the general U.S. population, though the heterogeneity found among this relatively disadvantaged sample highlights the importance of examining populations of youth who are at increased risk of firearm violence exposure.

Another limitation is that the linkage between the FFCWS and the GVA only allows for the study of deadly firearm incidents; thus, it is highly likely that we are underestimating the full impacts of spatially proximate firearm violence exposure, including nonfatal firearm injuries and shootings that do not result in injury. Additionally, because of limited sample size, we were unable to assess variation in the exposure and outcomes beyond the timeframe and geographic distance described, which may not align with youths' own perceptions of temporal, neighborhood, or community boundaries. Future analyses with larger samples of youth may allow for inspections of differential effects due to varying temporal or geographic proximity. Finally, qualitative research directly engaging youth, caregivers, and families is essential for more complete understanding of associations explored in this research.

## 5. Conclusion

This study furthers research on the impacts of community firearm violence exposure on youths' mental health and provides added support for examining both average and heterogeneous effects of such exposure. We illustrate the importance of conceptualizing community firearm violence exposure beyond direct or witnessed experience to account for the broader mental health-related harms felt by those who live and learn in neighborhoods where firearm violence occurs. Our results suggest that youth most at risk for spatially proximate firearm violence exposure, and particularly boys and young men, are in great need of intervention and prevention efforts that dually work to reduce exposure to community firearm violence while mitigating its mental health consequences.

## Supplementary Material

Additional details on analytic strategy and tables showing ancillary analyses results

## Figures and Tables

**Table 1:** Average effects of spatially proximate firearm homicide exposure on youths' depression and anxiety symptoms for full sample, boys, and girls.

	Unmatched						Matched					
	Total		Boys		Girls		Total		Boys		Girls	
Outcomes	b	Se	b	Se	b	Se	b	Se	b	Se	b	Se
Depression	0.075*	0.038	0.044	0.053	0.101^	0.055	0.113**	0.034	0.086	0.055	0.097	0.070
Anxiety	0.025	0.036	−0.044	0.050	0.081	0.053	0.060	0.041	0.041	0.069	0.081	0.072
Treatment N	1769		873		896		1718		852		863	
Control N	1317		709		608		1317		706		603	

Note: Unmatched results were yielded from multiple regression models, whereas matched results were generated from propensity score matching models.

^: p < .10

*: p < .05

**: p < .01

***: p < .001.

**Table 2a:** Descriptive statistics (percent/means and SE) of covariates by propensity score strata, full sample.

Variable	Stratum 1 p = [0–0.40)	Stratum 2 p = [0.40–0.60)	Stratum 3 p = [0.60–0.99)
Individual characteristics			
Girls	42.18%	47.57%	52.82%
Black	6.01%	47.06%	75.15%
White	56.20%	7.80%	1.00%
Latinx	24.03%	36.06%	19.87%
Age	15.32 (0.59)	15.52 (0.72)	15.61(0.73)
Impulsivity	2.33 (0.67)	2.48 (0.69)	2.56 (0.70)
Internalizing behavior	4.77 (4.90)	4.84 (5.05)	5.22 (6.20)
Externalizing behavior	4.92 (5.67)	5.72 (5.68)	7.26 (7.82)
Peer bully experience	0.48 (0.64)	0.59 (0.74)	0.68 (0.83)
Caregiver experienced intimate partner violence	0.45 (0.50)	0.37 (0.48)	0.39 (0.49)
Family characteristics			
Maternal education	2.84 (0.99)	2.12 (0.91)	1.78 (0.81)
Maternal depression	17.15%	16.50%	17.48%
Parents' tobacco use	35.29%	39.77%	52.36%
Parents' marijuana use	6.51%	9.85%	15.95%
Parents' alcohol use	89.36%	79.03%	72.16%
Community characteristics			
Caregiver witnessed weapon attack in community	0.02 (0.15)	0.03 (0.20)	0.24 (0.68)
Caregiver witnessed shooting in community	0.01 (0.08)	0.01(0.12)	0.17 (0.57)
Gangs are a problem in community	0.21 (0.45)	0.43 (0.63)	1.06 (1.01)
Neighborhood disadvantage	0.69 (0.57)	0.90 (0.68)	2.09 (1.19)
Low neighborhood collective efficacy	11.64 (3.54)	13.34 (4.38)	16.87 (5.87)

Note: Standard errors are shown in parentheses.

**Table 2b:** Descriptive statistics (percent/means and SE) of covariates by propensity score strata, boys only.

Variable	Stratum 1 p = [0–0.5)	Stratum 2 p = [0.5–0.6)	Stratum 3 p = [0.6–0.99)
Individual characteristics			
Girls	–	–	–
Black	0.00%	0.00%	91.08%
White	47.33%	38.21%	0.14%
Latinx	35.41%	51.79%	8.11%
Age	15.33 (0.59)	15.52 (0.80)	15.69 (0.72)
Impulsivity	2.44 (0.68)	2.38 (0.66)	2.58 (0.67)
Internalizing behavior	4.51 (4.71)	4.73 (5.16)	5.45 (6.66)
Externalizing behavior	5.46 (5.68)	5.79 (5.51)	8.44 (8.27)
Peer bully experience	0.44 (0.59)	0.51 (0.69)	0.77 (0.89)
Caregiver intimate partner violence experience	0.44 (0.50)	0.35 (0.48)	0.38 (0.49)
Family characteristics			
Maternal education	2.51 (1.09)	1.93 (0.93)	1.97 (0.85)
Maternal depression	17.26%	17.86%	17.84%
Parents' tobacco use	40.39%	40.00%	48.78%
Parents' marijuana use	4.45%	7.14%	18.78%
Parents' alcohol use	90.57%	74.29%	70.41%
Community characteristics			
Caregiver witnessed weapon attack in community	0.03 (0.21)	0.08 (0.34)	0.22 (0.66)
Caregiver witnessed shooting in community	0.00 (0.00)	0.0 (0.08)	0.16 (0.52)
Gangs are a problem in community	0.39 (0.68)	0.73 (0.92)	0.84 (0.96)
Neighborhood disadvantage	0.97 (0.83)	1.41 (1.08)	1.69 (1.27)
Low neighborhood collective efficacy	13.05 (4.68)	15.24 (5.54)	15.25 (5.82)

Note: Standard errors are shown in parentheses.

**Table 2c:** Descriptive statistics (percent/means and SE) of covariates by propensity score strata, girls only.

Variable	Stratum 1 p = [0–0.4)	Stratum 2 p = [0.4–0.6)	Stratum 3 p = [0.6–0.99)
Individual characteristics			
Girls	–	–	–
Black	5.38%	46.98%	72.81%
White	62.61%	8.52%	1.14%
Latinx	20.11%	36.54%	21.22%
Age	15.35 (0.67)	15.50 (0.63)	15.55 (0.72)
Impulsivity	2.27 (0.70)	2.41(0.70)	2.58 (0.69)
Internalizing behavior	4.81(4.81)	4.82 (4.50)	5.28 (6.09)
Externalizing behavior	4.22 (5.16)	5.07 (5.59)	6.52 (7.46)
Peer bully experience	0.50 (0.64)	0.55 (0.73)	0.66 (0.80)
Caregiver intimate partner violence experience	0.48 (0.50)	0.44 (0.50)	0.36 (0.48)
Family characteristics			
Maternal education	2.77 (0.99)	2.17 (0.96)	1.82 (0.83)
Maternal depression	17.00%	15.11%	17.41%
Parents' tobacco use	35.98%	43.41%	50.83%
Parents' marijuana use	8.50%	13.74%	13.21%
Parents' alcohol use	86.97%	81.04%	73.19%
Community characteristics			
Caregiver witnessed weapon attack in community	0.03 (0.20)	0.07 (0.31)	0.21 (0.65)
Caregiver witnessed shooting in community	0.01 (0.11)	0.02 (0.13)	0.18 (0.60)
Gangs are a problem in community	0.22 (0.45)	0.43 (0.63)	1.04 (0.01)
Neighborhood disadvantage	0.56 (0.37)	0.70 (0.56)	2.08 (1.16)
Low neighborhood collective efficacy	11.90 (3.68)	13.71 (4.62)	16.37 (5.86)

Note: Standard errors are shown in parentheses.

**Table 3a:** Heterogeneous effects of spatially proximate firearm homicide exposure on youths' depression and anxiety symptoms.

Full sample	Level 1						Level 2	
	Stratum 1 p = [0–0.4)		Stratum 2 p = [0.4–0.6)		Stratum 3 p = [0.6–0.99)		Trend	
				
Outcomes	b	Se	b	Se	b	Se	b	Se
Depression	−0.020	0.077	0.107^	0.064	0.132*	0.066	0.073	0.051
Anxiety	−0.031	0.077	−0.009	0.065	0.082	0.066	0.058	0.050
Treatment N	219		505		1045			
Control N	526		498		293			

Note:

^: p < .10

*: p < .05

**: p < .01

***: p < .001.

**Table 3b:** Heterogeneous effects of spatially proximate firearm homicide exposure on boys' depression and anxiety symptoms.

Boys	Level 1						Level 2	
	Stratum 1 p = [0–0.5)		Stratum 2 p = [0.5–0.6)		Stratum 3 p = [0.6–0.99)		Trend	
				
Outcomes	b	Se	b	Se	b	Se	b	Se
Depression	0.068	0.074	−0.058	0.094	0.218*	0.103	0.055	0.062
Anxiety	−0.026	0.073	−0.134	0.091	0.070	0.107	0.029	0.064
Treatment N	273		272		328			
Control N	350		213		146			

Note:

^: p < .10

*: p < .05

**: p < .01

***: p < .001.

**Table 3c:** Heterogeneous effects of spatially proximate firearm homicide exposure on girls' depression and anxiety symptoms.

Girls	Level 1						Level 2	
	Stratum 1 p = [0–0.4)		Stratum 2 p = [0.4–0.6)		Stratum 3 p = [0.6–0.99)		Trend	
Outcomes	b	Se	b	Se	b	Se	b	Se
Depression	0.120	0.109	0.025	0.103	0.127	0.095	0.008	0.072
Anxiety	0.125	0.117	0.013	0.010	0.101	0.094	−0.004	0.074
Treatment N	111		197		593			
Control N	254		218		131			

Note:

^: p < .10

*: p < .05

**: p < .01

***: p < .001.

**Table 4:** Mean comparisons of potential moderators in treatment (T) and control (C) groups and percent differences (PD) within each stratum, boys only.

Variables	Stratum 1			Stratum 2			Stratum 3		
	T	C	PD	T	C	PD	T	C	PD
Police stops									
Ever been stopped	0.265	0.297	10.774	0.426	0.372	14.516	0.427	0.416	2.644
Age stopped	12.583	12.667	0.663	12.877	13.049	1.318	13.122	12.797	2.540
Times stopped	2.661	4.000	33.475	2.338	2.429	3.746	3.404	3.240	5.062
Witness to police stops									
Witnessed in neighborhood	0.492	0.425	15.765	0.604	0.496	21.774^	0.660	0.530	24.528**
Witnessed in school	0.438	0.468	6.410	0.523	0.558	6.272	0.516	0.530	2.642
Know people stopped	0.533	0.557	4.309	0.619	0.619	0.000	0.624	0.602	3.654
Unjust treatment by police									
Witnessed/knew people treated unjustly	1.196	1.133	5.560	1.427	1.251	14.069^	1.571	1.346	16.716**
Personally treated unjustly	0.427	0.435	1.839	0.740	0.700	5.714*	0.779	0.683	14.056
Legal system involvement									
Ever been arrested	0.281	0.327	14.067	0.468	0.434	7.834	0.524	0.492	6.504
Ever been convicted	0.022	0.050	56.000	0.071	0.088	19.318	0.131	0.114	14.912
Ever been sentenced	0.032	0.052	38.462	0.078	0.097	19.588	0.159	0.119	33.613
Ever in juvenile detention before/during the hearing	0.016	0.047	65.957	0.065	0.088	26.136	0.142	0.108	31.481
Ever in juvenile detention following judge's decision	0.022	0.035	37.143	0.059	0.088	32.955	0.131	0.097	35.052
Age arrested	14.000	13.692	2.249	13.875	13.429	3.321	13.529	13.938	2.934
Times arrested	1.333	2.846	53.162	2.000	2.429	17.662	2.240	1.733	25.256
Mother/caregiver-child closeness	2.506	2.433	3.000	2.484	2.481	0.121	2.457	2.473	0.647
Mother/caregiver-child engagement	2.347	2.279	2.984	2.309	2.312	0.130	2.308	2.286	0.962
Connectedness at school	3.595	3.564	0.870	3.513	3.588	2.090	3.388	3.382	0.177
Extracurricular and community involvement	1.235	1.291	4.338	1.272	1.123	13.268	1.193	1.061	12.441^

Note: T represents treatment group, C represents control group, PD represents percent difference between treatment and control group.

^: p < .10

*: p < .05

**: p < .01

***: p < .001.

## Data Availability

The authors do not have permission to share data.

## Footnotes

1 We refer to these fatal firearm violence incidents as homicide incidents. However, the fatal firearm violence incidents linked to the FFCWS may include unintentional and undetermined firearm deaths, which nationally made up approximately 2.1% of annual deaths from firearms, on average, during the wave 6 data collection period, 2014–2017.
