# Supplementary material for: Heterogeneous effects of spatially proximate firearm homicide exposure on anxiety and depression symptoms among U.S. youth
Source: Prev Med. Author manuscript; Available in PMC 2023 Jul 31. (PMC10388845; doi:10.1016/j.ypmed.2022.107224)
Supplement: Additional details on analytic strategy and tables showing ancillary analyses results [file NIHMS1917548-supplement-Additional_details_on_analytic_strategy_and_tables_showing_ancillary_analyses_results.docx]

Supplementary Statistical Analysis Details

In this study, we examined average and heterogeneous associations between spatially proximate firearm homicide exposure and anxiety and depression among youth. We first estimated the average effects of firearm homicide exposure on anxiety and depression using covariate-adjusted ordinary least squares (OLS) regression, averaged across the imputed datasets, and then employed propensity score matching methods to calculate the predicted probability (propensity or risk) of exposure to spatially proximate firearm homicide for each observation and estimate the average effects of exposure on mental health for matched treated (exposed) youth. We applied Epanechnikov kernel matching with automatic bandwidth selection by cross-validation with respect to the mean of the propensity scores, matching treated youth with a weighted average of all controls with weights that were inversely proportional to the distance between the propensity scores of treated and control (unexposed) youth. The quality of matching was assessed to ensure that the distribution of covariates was balanced between treated and control youth.

Second, we estimated the heterogeneous relationship between spatially proximate firearm homicide exposure and youths’ mental health using heterogeneous treatment effect analysis with the stratification-multilevel method (Xie et al., 2012), which groups youth into propensity score strata. We determined the number of strata so as to allow for comparable numbers of youth in each stratum, with at least 20 each of treated and control youth (Perreira et al., 2005), and natural cut-points of the propensity scores (e.g., *p* = [0.00-0.40), *p* = [0.40-0.60), and *p* = [0.60-1.0)). Each stratum represents a different level of risk for experiencing at least 1 firearm homicide, ranging from low to high. Level 1 of the stratification-multilevel method allows for the estimation of stratum-specific effects of firearm homicide exposure on youths’ mental health within strata. At Level 2, we evaluated a linear trend across the strata using variance-weighted least squares regression of the stratum-specific exposure effects.

Third, to examine possible sources of heterogeneous exposure effects, we conducted ancillary analyses comparing the mean values of additional variables across treated and control youth within each propensity score stratum. These potential moderating variables included youths’ contact and treatment by law enforcement (frequency of police stops for oneself and others, age at first police stop, unjust treatment by police for oneself and others), criminal legal system involvement (frequency of arrests, age at first arrest, convictions, sentencings, juvenile detention), connectedness at home and school (closeness and engagement with mother or primary caregiver, connectedness to school), and extracurricular and community involvement (participation in sports, arts, clubs, religious services, volunteer activities). All potential moderators were measured at wave 6. For each possible moderator, statistically significant differences between treated and control youth were determined using two-tailed tests, and the magnitude of percent difference between treated and control youth was compared within and across strata to assess the strength of each variable in helping to explain heterogeneous exposure effects. Limitations in the data prevented precise determination of temporal ordering of events; thus, in this study, we could not perform formal tests of moderation. As such, these analyses were intended as exploratory and hypothesis-generating assessments of factors that may exacerbate risk or confer resilience among youth who may be differentially vulnerable to anxiety or depression as a result of spatially proximate firearm homicide exposure.

Supplementary Table 1a. Mean comparisons of potential moderators in treatment (T) and control (C) groups and percent difference (PD) within each stratum, full sample

| Variables | | Stratum 1 | | |  | Stratum 2 | | |  | Stratum 3 | | |
| --- | --- | --- | --- | --- | --- | --- | --- | --- | --- | --- | --- | --- |
|  |  | T | C | PD |  | T | C | PD |  | T | C | PD |
| Police stops | |  |  |  |  |  |  |  |  |  |  |  |
|  | Ever been stopped | 0.206 | 0.206 | 0.000 |  | 0.278 | 0.304 | 8.553 |  | 0.297 | 0.291 | 2.062 |
|  | Age stopped | 12.425 | 12.817 | 3.058 |  | 12.917 | 12.826 | 0.709 |  | 13.15 | 12.82 | 2.574 |
|  | Times stopped | 4.400 | 2.322 | 89.492^ |  | 2.609 | 3.162 | 17.489 |  | 2.888 | 2.449 | 17.926 |
| Witness to police stops | | | | | | | | | | | | |
|  | Witnessed in neighborhood | 0.437 | 0.372 | 17.473 |  | 0.485 | 0.442 | 9.729 |  | 0.633 | 0.509 | 24.361*** |
|  | Witnessed in school | 0.452 | 0.460 | 1.739 |  | 0.469 | 0.505 | 7.129 |  | 0.497 | 0.503 | 1.193 |
|  | Know people stopped | 0.533 | 0.520 | 2.500 |  | 0.545 | 0.566 | 3.710 |  | 0.601 | 0.568 | 5.810 |
| Unjust treatment by police | | | |  |  |  |  |  |  |  |  |  |
|  | Witnessed/knew people treated unjustly | 1.166 | 1.038 | 12.331^ |  | 1.256 | 1.222 | 2.782 |  | 1.562 | 1.340 | 16.567*** |
|  | Personally treated unjustly | 0.318 | 0.291 | 9.278 |  | 0.458 | 0.478 | 4.184 |  | 0.541 | 0.463 | 16.847 |
| Legal system involvement | | | |  |  |  |  |  |  |  |  |  |
|  | Ever been arrested | 0.217 | 0.216 | 0.463 |  | 0.315 | 0.347 | 9.222 |  | 0.360 | 0.338 | 6.509 |
|  | Ever been convicted | 0.016 | 0.020 | 20.000 |  | 0.050 | 0.065 | 23.077 |  | 0.087 | 0.062 | 40.323 |
|  | Ever been sentenced | 0.030 | 0.020 | 50.000 |  | 0.062 | 0.073 | 15.068 |  | 0.101 | 0.071 | 42.254 |
|  | Ever in juvenile detention before/during the hearing | 0.010 | 0.017 | 41.176 |  | 0.052 | 0.065 | 20.000 |  | 0.092 | 0.065 | 41.538 |
|  | Ever in juvenile detention following judge’s decision | 0.010 | 0.015 | 33.333 |  | 0.045 | 0.051 | 11.765 |  | 0.085 | 0.056 | 51.786 |
|  | Age arrested | 14.500 | 13.250 | 9.434 |  | 13.94 | 13.68 | 1.901 |  | 13.548 | 13.647 | 0.725 |
|  | Times arrested | 1.000 | 2.750 | 63.636 |  | 1.765 | 2.526 | 30.127 |  | 2.216 | 1.938 | 14.345 |
| Mother/caregiver-child closeness | | 2.494 | 2.441 | 2.172 |  | 2.420 | 2.396 | 1.002 |  | 2.378 | 2.394 | 0.669 |
| Mother/caregiver-child engagement | | 2.323 | 2.300 | 1.000 |  | 2.301 | 2.313 | 0.519 |  | 2.322 | 2.280 | 1.842 |
| Connectedness at school | | 3.520 | 3.573 | 1.483 |  | 3.468 | 3.438 | 0.873 |  | 3.341 | 3.384 | 1.271 |
| Extracurricular and community involvement | | 1.274 | 1.407 | 9.453^ |  | 1.200 | 1.195 | 0.418 |  | 1.208 | 1.127 | 7.187 |

Note: T represents treatment group, C represents control group, PD represents percent difference between treatment and control group.

^: p < .10; *: p < .05; **: p < .01; ***: p < .001

Supplementary Table 1b. Mean comparisons of potential moderators in treatment (T) and control (C) groups and percent differences (PD) within each stratum, girls only

| Variables | | Stratum 1 | | |  | Stratum 2 | | |  | Stratum 3 | | |
| --- | --- | --- | --- | --- | --- | --- | --- | --- | --- | --- | --- | --- |
|  |  | T | C | PD |  | T | C | PD |  | T | C | PD |
| Police stops | |  |  |  |  |  |  |  |  |  |  |  |
|  | Ever been stopped | 0.172 | 0.106 | 62.264^ |  | 0.142 | 0.208 | 31.731 |  | 0.186 | 0.189 | 1.587 |
|  | Age stopped | 12.530 | 13.556 | 7.569 |  | 12.826 | 12.571 | 2.028 |  | 13.272 | 12.806 | 3.639 |
|  | Times stopped | 2.500 | 1.714 | 45.858 |  | 1.885 | 2.771 | 31.974 |  | 2.257 | 2.125 | 6.212 |
| Witness to police stops | | | | | | | | | | | | |
|  | Witnessed in neighborhood | 0.414 | 0.336 | 23.214 |  | 0.437 | 0.372 | 17.473 |  | 0.589 | 0.479 | 22.965* |
|  | Witnessed in school | 0.438 | 0.462 | 5.195 |  | 0.448 | 0.457 | 1.969 |  | 0.482 | 0.485 | 0.619 |
|  | Know people stopped | 0.494 | 0.511 | 3.327 |  | 0.519 | 0.457 | 13.567 |  | 0.577 | 0.550 | 4.909 |
| Unjust treatment by police | | | |  |  |  |  |  |  |  |  |  |
|  | Witnessed/knew people treated unjustly | 1.124 | 1.025 | 9.659 |  | 1.269 | 1.129 | 12.400 |  | 1.519 | 1.269 | 19.701** |
|  | Personally treated unjustly | 0.276 | 0.148 | 86.486 |  | 0.228 | 0.331 | 31.118 |  | 0.330 | 0.306 | 7.843 |
| Legal system involvement | | | |  |  |  |  |  |  |  |  |  |
|  | Ever been arrested | 0.195 | 0.106 | 83.962* |  | 0.163 | 0.225 | 27.556 |  | 0.224 | 0.201 | 11.443 |
|  | Ever been convicted | 0.034 | 0.000 | -* |  | 0.027 | 0.023 | 17.391 |  | 0.051 | 0.012 | 325.000^ |
|  | Ever been sentenced | 0.046 | 0.000 | - * |  | 0.033 | 0.029 | 13.793 |  | 0.058 | 0.024 | 141.667 |
|  | Ever in juvenile detention before/during the hearing | 0.023 | 0.000 | -* |  | 0.033 | 0.023 | 43.478 |  | 0.055 | 0.018 | 205.556 |
|  | Ever in juvenile detention following judge’s decision | 0.023 | 0.000 | -* |  | 0.022 | 0.017 | 29.412 |  | 0.050 | 0.012 | 316.667^ |
|  | Age arrested | 13.000 | 15.000 | 13.333 |  | 13.833 | 12.500 | 10.664 |  | 13.652 | 13.333 | 2.393 |
|  | Times arrested | 1.000 | 2.000 | 50.000 |  | 1.167 | 2.750 | 57.564 |  | 2.240 | 2.667 | 16.011 |
| Mother/caregiver-child closeness | | 2.425 | 2.434 | 0.370 |  | 2.299 | 2.341 | 1.794 |  | 2.327 | 2.317 | 0.432 |
| Mother/caregiver-child engagement | | 2.318 | 2.331 | 0.558 |  | 2.272 | 2.313 | 1.773 |  | 2.333 | 2.285 | 2.101 |
| Connectedness at school | | 3.399 | 3.529 | 3.684* |  | 3.397 | 3.428 | 0.904 |  | 3.298 | 3.330 | 0.961 |
| Extracurricular and community involvement | | 1.368 | 1.507 | 9.224 |  | 1.208 | 1.338 | 9.716 |  | 1.190 | 1.139 | 4.478 |

Note: T represents treatment group, C represents control group, PD represents percent difference between treatment and control group.

^: p < .10; *: p < .05; **: p < .01; ***: p < .001

- PD not available due to denominator being 0

Supplementary Table 2a. Means of potential effect moderators for each stratum, and percent differences (PD) between each stratum, full sample

| Variables | | Stratum 1 | Stratum 2 | Stratum 3 | PD12 | PD23 | PD13 | |  |
| --- | --- | --- | --- | --- | --- | --- | --- | --- | --- |
| Police stops | |  |  |  |  |  |  | |  |
|  | Ever been stopped | 0.206 | 0.290 | 0.296 | 40.777*** | 2.069 | 43.689*** | |  |
|  | Age stopped | 12.719 | 12.872 | 13.079 | 1.203 | 1.608 | 2.830* | |  |
|  | Times stopped | 2.839 | 2.887 | 2.789 | 1.691 | 3.395 | 1.761 | |  |
| Witness to police stops | | | | |  |  |  | |  |
|  | Witnessed in neighborhood | 0.389 | 0.465 | 0.605 | 19.537** | 30.108*** | 55.527*** | |  |
|  | Witnessed in school | 0.458 | 0.486 | 0.498 | 6.114 | 2.469 | 8.734^ | |  |
|  | Know people stopped | 0.523 | 0.555 | 0.593 | 6.119 | 6.847^ | 13.384** | |  |
| Unjust treatment by police | |  |  |  |  |  |  | |  |
|  | Witnessed/knew people treated unjustly | 1.070 | 1.239 | 1.511 | 15.794*** | 21.953*** | 41.215*** | |  |
|  | Personally treated unjustly | 0.298 | 0.468 | 0.523 | 57.047*** | 11.752 | 75.503*** | |  |
| Legal system involvement | | | | | | | |  | |
|  | Ever been arrested | 0.216 | 0.330 | 0.355 | 52.778*** | 7.576 | 64.352*** | |  |
|  | Ever been convicted | 0.019 | 0.057 | 0.081 | 200.000** | 42.105^ | 326.316*** | |  |
|  | Ever been sentenced | 0.023 | 0.067 | 0.094 | 191.304** | 40.299 | 308.696*** | |  |
|  | Ever in juvenile detention before/during the hearing | 0.015 | 0.058 | 0.086 | 286.667 | 48.276^ | 473.333*** | |  |
|  | Ever in juvenile detention following judge’s decision | 0.014 | 0.048 | 0.079 | 242.857** | 64.583* | 464.286*** | |  |
|  | Age arrested | 13.500 | 13.806 | 13.467 | 2.267 | 2.455 | 0.244 | |  |
|  | Times arrested | 2.400 | 2.167 | 2.167 | 9.708 | 0 | 9.708 | |  |
| Mother/caregiver-child closeness | | 2.455 | 2.408 | 2.381 | 1.914^ | 1.121 | 3.014** | |  |
| Mother/caregiver-child engagement | | 2.306 | 2.306 | 2.313 | 0 | 0.304 | 0.304 | |  |
| Connectedness at school | | 3.560 | 3.454 | 3.351 | 2.978*** | 3.027*** | 5.871*** | |  |
| Extracurricular and community involvement | | 1.374 | 1.198 | 1.189 | 12.809*** | 0.754 | 13.464*** | |  |

Note: PD12 represents percent difference between Stratum 1 and Stratum 2; PD23 represents percent difference between Stratum 2 and Stratum 3; PD13 represents percent difference between Stratum 1 and Stratum 3

^: p < .10; *: p < .05; **: p < .01; ***: p < .001

Supplementary Table 2b. Means of potential effect moderators for each stratum, and percent differences (PD) between each stratum, boys only

| Variables | | Stratum 1 | Stratum 2 | Stratum 3 | PD12 | PD23 | PD13 | |  |
| --- | --- | --- | --- | --- | --- | --- | --- | --- | --- |
| Police stops | |  |  |  |  |  |  | |  |
|  | Ever been stopped | 0.287 | 0.403 | 0.424 | 40.418*** | 5.211 | 47.735*** | |  |
|  | Age stopped | 12.642 | 12.943 | 13.041 | 2.381 | 0.757 | 3.156* | |  |
|  | Times stopped | 3.048 | 2.374 | 3.362 | 22.113 | 41.618* | 10.302 | |  |
| Witness to police stops | | | | |  |  |  | |  |
|  | Witnessed in neighborhood | 0.446 | 0.558 | 0.646 | 25.112** | 15.771^ | 44.843*** | |  |
|  | Witnessed in school | 0.458 | 0.537 | 0.520 | 17.249* | 3.166 | 13.537* | |  |
|  | Know people stopped | 0.549 | 0.619 | 0.619 | 12.751^ | 0 | 12.750* | |  |
| Unjust treatment by police | |  |  |  |  |  |  | |  |
|  | Witnessed/knew people treated unjustly | 1.152 | 1.354 | 1.512 | 17.535** | 11.669* | 31.25*** | |  |
|  | Personally treated unjustly | 0.433 | 0.647 | 0.754 | 49.423*** | 16.538 | 74.134*** | |  |
| Legal system involvement | | | | | | | |  | |
|  | Ever been arrested | 0.312 | 0.453 | 0.515 | 45.192*** | 13.687 | 65.064*** | |  |
|  | Ever been convicted | 0.041 | 0.079 | 0.126 | 92.683^ | 59.494 | 207.317*** | |  |
|  | Ever been sentenced | 0.046 | 0.086 | 0.149 | 86.957^ | 73.256^ | 223.913*** | |  |
|  | Ever in juvenile detention before/during the hearing | 0.037 | 0.075 | 0.133 | 102.703^ | 77.333^ | 259.460*** | |  |
|  | Ever in juvenile detention following judge’s decision | 0.031 | 0.071 | 0.122 | 129.032* | 71.831^ | 293.548*** | |  |
|  | Age arrested | 13.750 | 13.667 | 13.627 | 0.604 | 0.293 | 0.895 | |  |
|  | Times arrested | 2.563 | 2.200 | 2.123 | 14.163 | 3.500 | 17.167 | |  |
| Mother/caregiver-child closeness | | 2.456 | 2.483 | 2.461 | 1.099 | 0.886 | 0.204 | |  |
| Mother/caregiver-child engagement | | 2.301 | 2.310 | 2.302 | 0.391 | 0.346 | 0.043 | |  |
| Connectedness at school | | 3.573 | 3.545 | 3.387 | 0.784 | 4.457*** | 5.206*** | |  |
| Extracurricular and community involvement | | 1.273 | 1.209 | 1.159 | 5.027 | 4.136 | 8.955* | |  |

Note: PD12 represents percent difference between Stratum 1 and Stratum 2; PD23 represents percent difference between Stratum 2 and Stratum 3; PD13 represents percent difference between Stratum 1 and Stratum 3

^: p < .10; *: p < .05; **: p < .01; ***: p < .001

Supplementary Table 2c. Means of potential effect moderators for each stratum, and percent differences (PD) between each stratum, girls only

| Variables | | Stratum 1 | Stratum 2 | Stratum 3 | PD12 | PD23 | PD13 | |  |
| --- | --- | --- | --- | --- | --- | --- | --- | --- | --- |
| Police stops | |  |  |  |  |  |  | |  |
|  | Ever been stopped | 0.122 | 0.174 | 0.187 | 42.623^ | 53.279** | 7.471 | |  |
|  | Age stopped | 13.190 | 12.672 | 13.172 | 3.927 | 0.136 | 3.946^ | |  |
|  | Times stopped | 1.976 | 2.393 | 2.228 | 21.103 | 12.753 | 6.895 | |  |
| Witness to police stops | | | | |  |  |  | |  |
|  | Witnessed in neighborhood | 0.355 | 0.406 | 0.556 | 14.366 | 59.437*** | 39.409*** | |  |
|  | Witnessed in school | 0.456 | 0.452 | 0.482 | 0.877 | 5.702 | 6.637 | |  |
|  | Know people stopped | 0.507 | 0.489 | 0.571 | 3.550 | 12.623* | 16.769** | |  |
| Unjust treatment by police | |  |  |  |  |  |  | |  |
|  | Witnessed/knew people treated unjustly | 1.050 | 1.201 | 1.464 | 14.381* | 39.429*** | 21.898*** | |  |
|  | Personally treated unjustly | 0.180 | 0.278 | 0.324 | 54.444* | 80.000*** | 16.547 | |  |
| Legal system involvement | | | | | | | |  | |
|  | Ever been arrested | 0.128 | 0.194 | 0.220 | 51.563* | 71.875** | 13.402 | |  |
|  | Ever been convicted | 0.009 | 0.025 | 0.043 | 177.778 | 377.778* | 72.000 | |  |
|  | Ever been sentenced | 0.011 | 0.031 | 0.051 | 181.818 | 363.636* | 64.516 | |  |
|  | Ever in juvenile detention before/during the hearing | 0.006 | 0.028 | 0.047 | 366.667^ | 683.333** | 67.857 | |  |
|  | Ever in juvenile detention following judge’s decision | 0.006 | 0.020 | 0.042 | 233.333 | 600.000** | 110.000 | |  |
|  | Age arrested | 14.000 | 13.300 | 13.615 | 5.000 | 2.750 | 2.368 | |  |
|  | Times arrested | 1.500 | 1.800 | 2.286 | 20.000 | 52.400 | 27.000 | |  |
| Mother/caregiver-child closeness | | 2.432 | 2.319 | 2.325 | 4.646** | 4.400** | 0.259 | |  |
| Mother/caregiver-child engagement | | 2.328 | 2.292 | 2.323 | 1.546 | 0.215 | 1.353 | |  |
| Connectedness at school | | 3.497 | 3.413 | 3.305 | 2.402* | 5.490*** | 3.164** | |  |
| Extracurricular and community involvement | | 1.473 | 1.271 | 1.179 | 13.714** | 19.959*** | 7.238^ | |  |

Note: PD12 represents percent difference between Stratum 1 and Stratum 2; PD23 represents percent difference between Stratum 2 and Stratum 3; PD13 represents percent difference between Stratum 1 and Stratum 3

^: p < .10; *: p < .05; **: p < .01; ***: p < .001
